# Hypoglycemic, Hypolipidemic, and Anti-Inflammatory Effects of Beta-Pinene in Diabetic Rats

**DOI:** 10.1155/2022/8173307

**Published:** 2022-05-17

**Authors:** Enaide Soares Santos, Geraldo Lucas Abrantes Coelho, Yan Kauê Saraiva Fontes Loula, Bárbara Lourenço Saraiva Landim, Cícera Norma Fernandes Lima, Sara Tavares de Sousa Machado, Maria Janice Pereira Lopes, Acléssia Damiana Soares Gomes, José Galberto Martins da Costa, Irwin Rose Alencar de Menezes, Henrique Douglas Melo Coutinho, Bonglee Kim, Cícero Francisco Bezerra Felipe, Samya de Araújo Neves, Marta Regina Kerntopf

**Affiliations:** ^1^Regional University of Cariri, URCA, Crato, Brazil; ^2^Faculty of Medicine Estacio of Juazeiro do Norte, FMJ, Juazeiro do Norte, Brazil; ^3^Department of Pathology, College of Korean Medicine, Kyung Hee University, Seoul 02447, Republic of Korea; ^4^Federal University of Paraíba, UFPB, João Pessoa, Brazil

## Abstract

**Background:**

Diabetes is a metabolic disease linked to multiple comorbidities, such as low-grade inflammation. *β*-pinene, a monoterpene commonly found in aromatic plants, is endowed with anti-inflammatory effect and this fact lead us to investigate the possible hypoglycemic, hypolipidemic and anti-inflammatory effects of the monoterpene in the alloxan-induced diabetes experimental model.

**Methods:**

Male Wistar rats (200–250 g) were treated orally with *β*-pinene (25, 50, 100, and 200 mg/kg) or glibenclamide (5 mg/kg), for seven consecutive days. Diabetes was induced by alloxan (40 mg/kg) through the penile vein. On the seventh day of treatment, blood samples were collected for biochemical analysis. The anti-inflammatory effect of *β*-pinene was evaluated using the carrageenan-induced paw edema model, followed by the carrageenan-induced peritonitis.

**Results:**

The treatment with *β*-pinene decreased plasma glucose, triglyceride, VLDL, LDL, and HDL levels, when compared to those of the control group. In addition, the association *β*-pinene 10 mg/kg + glibenclamide 2 mg/kg significantly decreased blood glucose, total cholesterol, and triglyceride level. Finally, oral treatment with *β*-pinene reduced carrageenan-induced paw edema and leukocyte migration in the peritoneum. Taken together, our results indicate that *β*-pinene shows hypoglycemic and hypolipemic effects, which may involve some common mechanisms of glibenclamide. Besides, the monoterpene presented an anti-inflammatory action in diabetic rats that needs further investigation in order to clarify such effect and its correlation with the alterations observed in plasma parameters of *β*-pinene-treated diabetic rats.

## 1. Introduction

Diabetes mellitus (DM) is a metabolic disease that promotes plasma hyperglycemia, secondary to reduced insulin secretion and/or resistance to its peripheral action [1]. According to the World Health Organization (WHO) [2], the classification of DM falls into 3 clinical classes: type 1 DM (DM1), type 2 DM (DM2), gestational DM, and other specific types of DM [3]. DM1 is characterized by the destruction of beta cells that eventually leads to the stage of absolute insulin deficiency. The destruction of beta cells is usually triggered by an autoimmune process, which can be detected by circulating autoantibodies, such as anti-glutamic acid decarboxylase (anti-GAD) and anti-islets and anti-insulin autoantibodies. Moreover, this type of DM is sometimes linked to other autoimmune diseases, such as Hashimoto's thyroiditis, Addison's disease, and myasthenia gravis [4]. On the other hand, DM2 is characterized by a deficiency or failure in maintaining glucose homeostasis, resulting from defects in insulin secretion and action, associated with a sedentary lifestyle and poor dietary habits. A family history of DM is also a risk factor for microvascular complications, kidney insufficiency, blindness, and cardiovascular disease [1]. Gestational DM is another type of DM, diagnosed during pregnancy [5], which similarly to DM2, is associated with both insulin resistance and decreased pancreatic beta-cell function. Lastly, other specific types of DM constitute a 4^th^ classification of DM that can be reported for a range of factors, including genetic defects in beta cell function and genetic defects in the insulin action as well as exocrine pancreas diseases, induced by drugs, chemical agents, or infections [3].

Currently, DM still remains a serious public health problem. It is associated worldwide with high rates of morbidity and mortality due to complications related to DM and dyslipidemia and to alterations that involve renal and hepatic oxidative damage and that worsen the clinical picture and increase mortality rates [6]. One way to avoid such complications is to maintain healthy lifestyle habits such as adequate nutrition, regular physical exercise, and new therapeutic alternatives that can control blood glucose [7].

The search for medicinal plants capable of treating diseases has awakened researchers and the pharmaceutical industry to invest more in new drugs endowed with relevant therapeutic action [8]. Indeed, the therapeutic action attributed to the presence of some biologically active molecules, such as flavonoids, alkaloids, terpenes, and tannins, can be proved through preclinical tests. For this reason, these substances have been the subject of incessant studies in multidisciplinary areas, with the aim of enriching the knowledge surrounding natural products as effective and safer sources of drugs derived from secondary metabolites [9].

Terpenes are substances derived from a plant's secondary metabolism and are classified based on the number of a five-carbon molecule, the isoprene unit, as follows: hemiterpenes (5C), monoterpenes (10C), sesquiterpenes (15C), diterpenes (20C), sesterterpenes (25C), triterpenes (30C), tetraterpenes (35C), and polyisoprenoids (>35 carbons). *β*-pinene is a cyclic monoterpene commonly found in essential oils from various aromatic medicinal plants [10] and presents important biological properties, such as myorelaxant, antimicrobial, antidepressant, antispasmodic, anti-inflammatory, anxiolytic, anticonvulsant, and hypotensive effects [11]. In addition, it has been reported that *β*-pinene may act as a hypoglycemic agent given its regenerating capacity in pancreatic beta cells [12]. Thus, in view of the reported *β*-pinene-mediated anti-inflammatory effects and the involvement of inflammation in DM, we sought to investigate the possible hypoglycemic, hypolipidemic, and anti-inflammatory effects of *β*-pinene in diabetic rats.

## 2. Methods

### 2.1. Drugs and Dose of Treatments

**Figure 1:**
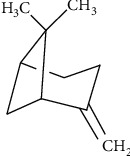
Chemical structure of *β*-pinene (Sigma-Aldrich, 2018).

Beta-pinene (Sigma Chemical Co., St. Louis, MO, USA), batch: MKBP3888V, was sponsored by the Regional University of Cariri-URCA (Figure 1). In the present work, we tested doses of the terpene that corresponded to values lower than 4% of its LD_50_ (LD_50_ > 5000 mg/kg, according to the provider). Glibenclamide (5 mg/kg) was purchased from Biosynthetic Laboratory (São Paulo, SP, Brazil) and used as control. Alloxan (40 mg/kg) and Tween-80 (2% v/v) were purchased from Sigma Chemical Co. (St. Louis, MO, USA). All other substances were donated by the Faculty of Medicine of Juazeiro do Norte.

### 2.2. Animals, Treatments, and Ethical Aspects

This study was carried out in the Laboratory of Biophysiology of the Faculty of Medicine of Juazeiro do Norte, and it was approved by the Faculty's Ethics Committee on the Use of Animals, under the process no. 2018.01.018. A total of 150 adult male Wistar rats (200 to 250 g) were donated from the vivarium of The Faculty of Medicine of Juazeiro do Norte. The animals were housed in groups of five, for at least 24 h prior to the experiments, in plastic boxes, receiving water and food ad libitum in a controlled environment with a 12 h light/dark cycle and regulated temperature (22 ± 2°C) and humidity (70%).

Alloxan (40 mg/kg, iv) was injected in male Wistar rats (250 g) and after 48 h, animals were subjected to blood collection for measurements of blood glucose (mg/dL). Only those showing glycemia levels higher than 250 mg/dL were submitted to the study. After this period, the animals were administered daily doses of 25, 50 and 100 mg/kg of *β*-pinene (BP) and glibenclamide (Glib 5 mg/kg) orally for 7 days. Untreated diabetic controls (DC) received vehicle. After this period, the animals underwent fasting and blood collection for biochemical analysis. At the end of the experiment, the animals were euthanized with sodium thiopental. A lethal dose of thiopental (150 mg/kg) and lidocaine (10 mg/mL) was used intraperitoneally [13].

### 2.3. Biochemical Analysis

Blood samples were centrifuged at 3000 rpm for 10 min to obtain the serum, which was used to determine glucose, total cholesterol (TC), HDL, LDL, and triglycerides (TG) levels and AST and ALT activities by standard enzymatic colorimetric methods, according to the manufacturer's instructions (Labtest®, Brazil).

### 2.4. Drug Association Studies

To investigate the possible mechanism of action of *β*-pinene, rats were treated for seven consecutive days with vehicle or *β*-pinene (10 mg/kg) or glibenclamide (2 mg/kg) or the association of *β*-pinene (10 mg/kg) plus glibenclamide (2 mg/kg). On the last day (7^th^ day), blood samples were collected to determine the blood glucose, cholesterol, and triglyceride levels.

### 2.5. Evaluation of the Anti-Inflammatory Effects of *β*-Pinene in Diabetic Rats

On the last day of treatment, 1 h after the drug's administration, an intraplantar injection of 40 *µ*L/paw of a 1% w/v carrageenan solution was given to the right hind paw. Paw volumes were then measured at intervals of 1, 2, 3, 4, and 24 h (standard times), for atypical progressive edema evaluation, which occurs in diabetic rats. The edema volume (EV) resulting from an acute inflammatory reaction was determined by the difference between the final (FV) and initial (IV) paw volumes [14].

### 2.6. Carrageenan-Induced Peritonitis

Diabetic rats were orally treated with vehicle, glibenclamide (5 mg/kg), dexamethasone (5 mg/kg), and *β*-pinene (50 mg/kg). After 60 min of treatment, each rodent received a 1% intraperitoneal injection of carrageenan. The fifth group consisted of healthy animals that did not receive any substance. After 4 h, a 3 mL volume of heparinized PBS was administered into the peritoneal cavity of the animals. Then, a gentle massage was performed in the abdominal region of the mouse and the peritoneal fluid was collected for a subsequent leukocyte count, which was performed by using the ABX Micros 60 device [15].

### 2.7. Statistical Analysis

The results that presented a normal distribution were represented as mean ± standard error of the mean (SEM) and analyzed by one-way or two-way analysis of variance (ANOVA) followed by the Bonferroni test. The results were considered significant at *p* < 0.05.

## 3. Results

### 3.1. Evaluation of Biochemical Parameters

Oral administration of *β*-pinene significantly decreased blood glucose, VLDL, HDL, and LDL levels (Table 1). On the other hand, no significant alterations were seen in the blood level of TC or in ALT and AST activities.

### 3.2. Drug Association Study: Evaluation of Acute Treatment Effects on Plasma Glycemic, Cholesterol, and Triglyceride Levels of Diabetic Rats

**Figure 2:**
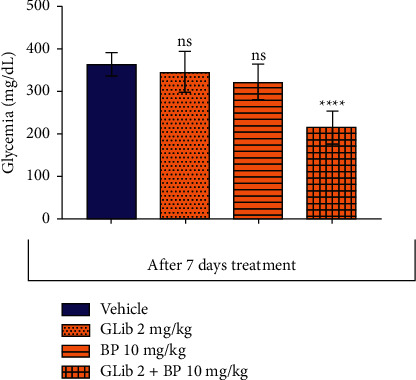
Effect of *β*-pinene plus glibenclamide 7-day oral treatment on the blood glucose level in diabetic rats. The columns represent the mean ± standard error of the mean (SEM), analyzed through two-way ANOVA and followed by Bonferroni (*n* = 10 mice per group). The values were considered significant at ^*∗∗*^*p* < 0.01; ^*∗∗∗*^, *p* < 0.001, ^*∗∗∗∗*^*p* < 0.0001 vs. vehicle.

The association of glibenclamide (2 mg/kg) plus *β*-pinene (10 mg/kg), when administered for 7 days, significantly decreased the blood glucose level by 41.6% (215.9 ± 38.37, *p* < 0.0001), when compared to the vehicle (365.1 ± 27.51). On the other hand, such effect was not observed in groups that were treated with *β*-pinene alone, at 10 mg/kg (323.3 ± 41.11) and glibenclamide alone, at 2 mg/kg (347.6 ± 48.5), when compared to the vehicle (Figure 2).

**Figure 3:**
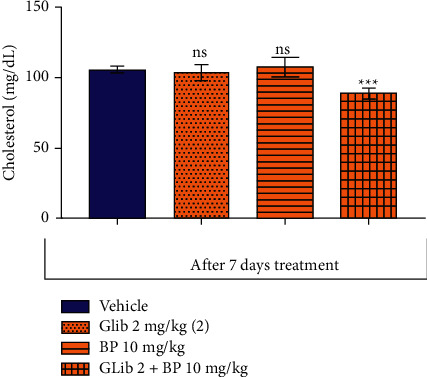
Effect of *β*-pinene plus glibenclamide 7-day oral treatment on the blood cholesterol level in diabetic rats. The columns represent the mean ± standard error of the mean (SEM), analyzed through two-way ANOVA followed by Bonferroni (*n* = 10 mice per group). The values were considered significant at ^*∗∗*^*p* < 0.01; ^*∗∗∗*^*p* < 0.001, ^*∗∗∗∗*^*p* < 0.0001 vs. vehicle.

**Figure 4:**
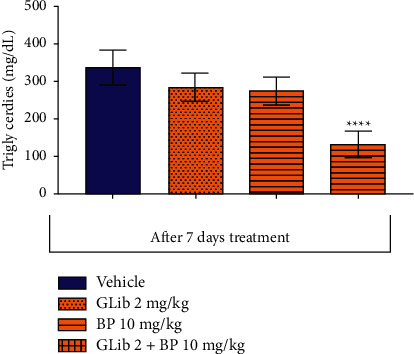
Effect of *β*-pinene plus glibenclamide 7-day oral treatment on the blood triglycerides level in diabetic rats. The columns represent the mean ± standard error of the mean (SEM), analyzed through two-way ANOVA followed by Bonferroni (*n* = 10 mice per group). The values were considered significant at ^*∗∗*^*p* < 0.01; ^*∗∗∗*^*p* < 0.001, ^*∗∗∗∗*^*p* < 0.0001 vs. vehicle.

The drug combination also promoted a significant reduction in cholesterol and triglyceride levels by 16.1% (105.8 ± 4.3, *p* < 0.001) and 29.1% (132.3 ± 34.96, *p* < 0.0001), respectively, when compared to the respective control groups (105.9 ± 2.7), and (338.1 ± 47.86) (Figures 3 and 4).

### 3.3. Acute Anti-Inflammatory Activity Evaluation in Rats with Alloxan-Induced Diabetes: Paw Edema

**Figure 5:**
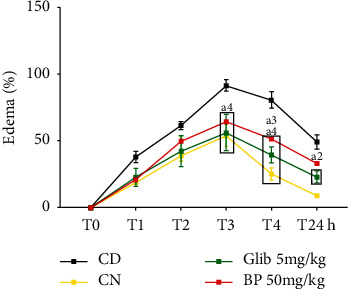
Effect of *β*-pinene on diabetic rats subjected to carrageenan-induced paw edema. Glib: glibenclamide; BP: *β*-pinene; CD: diabetic control; CN: normal control. The comparison of procedure value between diabetic groups and the normal group is considered significant at ^*∗∗*^*p* < 0.01; ^*∗∗∗*^*p* < 0.001, ^*∗∗∗∗*^*p* < 0.0001 vs. CD.

The seven day consecutive oral treatment with *β*-pinene (50 mg/kg) and glibenclamide (5 mg/kg) significantly reduced the edema at the 3^rd^ h of evaluation, by 29.6% (*p* < 0.001) and 38.4% (*p* < 0.001), respectively, when compared to the diabetic group (91.66 ± 4.3). In addition, *β*-pinene at 50 significantly reduced the edema at the 4^th^ h of evaluation by 36% (*p* < 0.001) and 26.8% (*p* < 0.001) when compared to the diabetic (80.8 ± 6.1) and normal control (25.3 ± 4.5) groups. Glibenclamide at 5 mg/kg also reduced paw edema by 68.6% (*p* < 0.001). After 24 h of edema induction, *β*-pinene at 50 reduced paw edema by 32.7% (*p* < 0.001) and 43.9% (*p* < 0.001), respectively, when compared to the diabetic (49.2 ± 5.3) and normal control (9.2 ± 1.3) groups. It should be noted that this time point (T24), represents the 7^th^ and last day of experiments. During this period, the normal control group presented a regression in the animal's paw edema, returning to a volume similar to that observed during the 1^st^ h of edema induction (Figure 5).

### 3.4. Carrageenan-Induced Peritonitis

**Figure 6:**
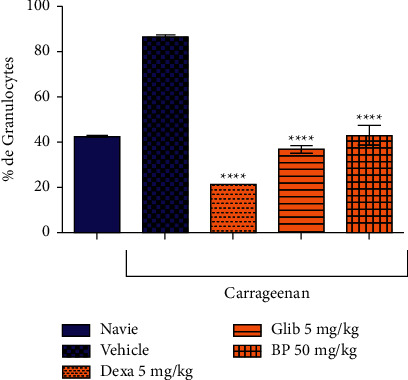
Effect of *β*-pinene on granulocyte migration in the carrageenan-induced peritonitis test. Columns represent the mean ± standard error of the mean (SEM), analyzed using two-way ANOVA followed by Bonferroni (*n* = 10 rats per group). Values were considered significant when ^*∗∗*^*p* < 0.01; ^*∗∗∗*^*p* < 0.001, ^*∗∗∗∗*^*p* < 0.0001 vs. vehicle.

**Figure 7:**
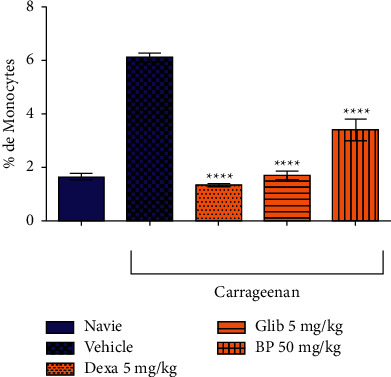
Effect of *β*-pinene on monocytes migration in the carrageenan-induced peritonitis test. Columns represent the mean ± standard error of the mean (SEM), analyzed using two-way ANOVA followed by Bonferroni (*n* = 10 rats per group). Values were considered significant when ^*∗∗*^*p* < 0.01; ^*∗∗∗*^*p* < 0.001, ^*∗∗∗∗*^*p* < 0.0001 vs. vehicle.

**Figure 8:**
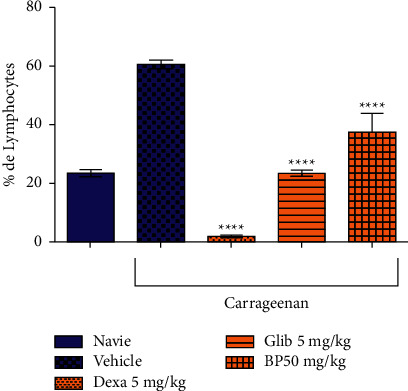
Effect of *β*-pinene on lymphocyte migration in the carrageenan-induced peritonitis test. Columns represent the mean ± standard error of the mean (SEM), analyzed using two-way ANOVA followed by Bonferroni (*n* = 10 rats per group). Values were considered significant when ^*∗∗*^*p* < 0.01; ^*∗∗∗*^*p* < 0.001, ^*∗∗∗∗*^*p* < 0.0001 vs. vehicle.

According to Figure 6, *β*-pinene significantly reduced leukocyte migration. It reduced granulocytes by 50.1% compared to the control group (86.6 ± 1.57). Similarly, it significantly reduced monocyte migration by 44.4% and lymphocyte migration by 37.9% compared to the control group (6.12 ± 0.3) and (60.8 ± 3.1), respectively (Figures 7 and 8).

Dexamethasone used as the standard drug in the anti-inflammatory effect significantly reduced the migration of granulocytes, monocytes, and lymphocytes in 75.3%, 77.7%, and 96.7%, respectively, in relation to the vehicle-treated group. The oral hypoglycemic (Glib 5 mg/kg), also showed a significant reduction of leukocyte migration in 18.5% in granulocytes, 71.8% in monocytes, and 61.3% in lymphocytes in the control group.

## 4. Discussion

*β*-pinene is a monoterpene that is found in essential oils as the enantiomers (+)-*β*-pinene and (−)-*β*-pinene [12]. It is known that some terpenes have the ability to maintain *β* cell performance and to decrease plasma hyperglycemia, in addition to other biological effects [16]. Such effects are particularly useful for the management of DM, which is a complex disease, as it involves interactions between genetic and environmental factors [17, 18]. The worldwide concern with chronic diseases, and in particular with DM, has stimulated an intensive search on aspects associated with its pathophysiology, aiming to improve early detection, prevention, and even treatment of associated complications [6]. Alloxan is an animal model for the study of experimental diabetes in rats [19, 20] as well as potential antidiabetic drugs. According to Sociedade Portuguesa de Diabetologia [21], alloxan does not destroy all pancreatic *β* cells despite causing DM2, it rather triggers a reduction in these cells. Thus, in the present study, we decided to use this method of inducing DM to test the hypoglycemic and hypolipidemic activities of *β*-pinene in rats.

Our results showed that *β*-pinene reduced glycemic levels in alloxan-induced diabetes in rats. Kahn et al. [22] report that some terpenes *β*-pinene and *α*-pinene have hypoglycemic effects in streptozotocin-treated mice. In addition, D'Adamo and Caprio [23] showed that two triterpenes led to a marked reduction in glucose levels in streptozotocin-induced diabetic rats. Salehi et al. [24] also showed that *Origanum vulgare* L. has hypoglycemic effects in streptozotocin-induced diabetic rats, possibly by the presence of *α*-pinene, a *β*-pinene isomer in the composition of the plant. Although the authors used a distinct diabetogenic agent, these findings contribute to the statement that chemical components present in plants are responsible for their pharmacological action. The data obtained from these authors also reinforces the fact that the isolated terpenic substances have similar effects on the glycemic level.

Menezes et al. [25] reported that species from the Fabaceae family contain several chemical substances in their composition, including *α*- and *β*-pinene, which caused a significant reduction in serum TG, TC, and high-density lipoprotein (HDL) levels in hyperglycemic rats treated with aqueous, ethanolic, and hexane leaf extracts from species belonging to this family. According to Menezes et al. [26], diabetes promotes changes in plasma lipid concentration. Hypertriglyceridemia is a common feature in patients with diabetes, as a result of both overproduction of VLDL by the liver, and diminished removal of triglyceride-rich lipoprotein, caused by reduced lipoprotein lipase (PLP) activity, the rate-limiting enzyme in triglyceride-rich lipoprotein metabolism. Deficiencies in LPL play an important role in the pathophysiology of hyperlipidemia in diabetes [27] and the treatment with insulin increases PLP activity thus causing a fall in plasma triglycerides [28]. In the present study, alloxan administration caused an increase in serum triglycerides and VLDL levels and the treatment with *β*-pinene reduced such biochemical parameters (Table 1), with the latter effect possibly involving an increase in the activity of LPL on hepatic and adipose tissues. Lucchesian et al. [29] attribute the reduction in the blood lipid profile of animals treated with *Syzygium cumini* L., to the plant's chemical constituents, including triterpenes.

Changes in the metabolism of plasma lipoproteins in patients with diabetes mellitus are quite common [30]. Valdes et al. [31] report that the *Rosmarinus officinalis* L. leaf extract has terpenic compounds, including *β*-pinene, which promoted a reduction of abdominal fat in mice by acting on a lipase, which breaks down fat, possibly due to the action of its chemical constituents, facilitating the reduction of total cholesterol levels. In our study, *β*-pinene showed no reduction in TC levels. As *β*-pinene presented some effects that resemble those of glibenclamide, we investigated whether the monoterpene shares the same mechanism of action as the sulfonylurea. Thus, lower doses of glibenclamide (2 mg/kg) and *β*-pinene (10 mg/kg) were tested alone and then in combination. The association of the two substances led to a reduction in glucose, cholesterol, and triglyceride levels in the blood of diabetic rats, while the use of both substances alone did not modify such biochemical parameters. Briefly, glibenclamide stimulates insulin secretion by blocking ATP-dependent K^+^ channels in pancreatic *β* cell membranes, causing depolarization and Ca^2+^ influx [32]. The results indicate that a possible synergism may be occurring between the two compounds and that the hypoglycemic action of *β*-pinene may present a mechanism like that of glibenclamide.

Based on the involvement of inflammation with diabetes and with strong evidence that *β*-pinene has anti-inflammatory effects, we investigated the anti-inflammatory effect of *β*-pinene in diabetic animals. Carrageenan is a sulfated polysaccharide obtained from some red algae species that promotes different inflammatory stimuli and recruits several inflammatory mediators to the injured site [33]. Briefly, carrageenan stimulates the release of vasoactive amines and promotes the latter release of cytokines (IL-1 and TNF-*α*) and prostaglandins PGE2 [24], followed by cell migration and plasma exudation [34]. This edema is proportional to the intensity of the inflammatory response and constitutes a useful parameter to determine the anti-inflammatory potential of a drug [35]. Carrageenan-induced paw edema was assessed in our study to evaluate the anti-inflammatory effect of *β*-pinene in diabetic rats. Our results showed that non-diabetic animal animals presented an edema elevation peak in the 3^rd^ h of inflammation, which later regressed to baseline levels within 24 h. This can be explained through studies by Wold [36] that show that inflammation reaches a peak in the first 3 h of edema induction, with this edema later decreasing to baseline levels when there are no new stimuli. However, diabetic rats have a different pattern from the normal ones.

As DM is a chronic disease, the entire inflammatory process has a longer time period. This is observed in the results presented in this study, in which untreated diabetic rats showed that edema lasted longer, until the 24^th^ h of the experiment. On the other hand, *β*-pinene reduced edema volume by up to 29.6% in the 3^rd^ h, when used at 50 mg/kg. Moreover, after 24 h, *β*-pinene also reduced edema by 32.7% at 50 mg/kg. Pioneering studies by Di Rosa [31] have previously shown that biogenic amines, responsible for vascular events, participate in the first phase of inflammation. In the 2^nd^ phase, evidenced only after 3 h of induction, cytokines, prostaglandins, and NO participate in the inflammatory process, further increasing the inflammatory reaction. Thus, *β*-pinene may possibly act by inhibiting inflammatory mediators that participate in the 2^nd^ phase of the process; however, more studies are necessary to test such a hypothesis.

Carrageenan-induced peritonitis is a model that involves cell migration and plasma exudation into the peritoneal cavity during an inflammatory process [37]. Neutrophils (polymorphonuclear), as well as macrophages, are the major phagocytic cells of the immune system. During the inflammatory process, the neutrophils are recruited to the inflammatory focus playing a defensive role to the organism. This model evaluates an acute inflammation, allowing quantification of leukocytes that migrate to the peritoneal cavity. This event is determined by the action of chemotactic mediators as cytokines (TNF-alpha, interleukin (IL)-1*β*, and IL-6), nitric oxide, peptide fragments of the complement system (C3a, C4a, and C5a), histamine, leukotrienes, prostaglandins, thromboxane, and PAF52 [38].

De Carvalho et al. [39] indicated that the activity of phenolic compounds is involved in the inhibition of inflammatory mediators, as well as the study in question that presents these compounds and shows a possible reduction in the expression of these cells evidenced in the peritonitis model. Some authors have also shown that medicinal plants rich in diterpenes, sesquiterpenes, and monoterpenes have anti-inflammatory and hypoglycemic effects [40]. Moller [41] attributed the analgesic and anti-inflammatory effects of ginger to the presence of gingerol. Carvalho et al. [40] evaluated the anti-inflammatory effect of *Bumelia sartorum* Mart. The aqueous extract, which is rich in terpenes, used the carrageenan-induced paw edema method in diabetic rats and reported a significant reduction in edema. This finding is similar to those obtained in our study, as the authors used a substance belonging to the terpene class and analyzed its anti-inflammatory activity using the same method. Nonetheless, although the considerations reported in this experiment show that there is an increase in the inflammatory process in diabetic rats when compared to normal ones, these findings cannot elucidate the exact mechanism by which *β*-pinene reduces edema in this model and further studies in this field are needed.

## 5. Conclusions

The oral administration of *β*-pinene, for seven consecutive days, presented hypoglycemic and hypolipidemic effects, which seems to involve, at least in part, the blocking of ATP-dependent K^+^ channels and the activation of LPL, respectively. The treatment with *β*-pinene also revealed an important anti-inflammatory effect, possibly by inhibiting inflammatory mediators that participate in the 2^nd^ phase of the process, possibly by decreasing leukocyte migration, indicating that its action may be linked to the inhibition of cytokine production. It is important to stress that other studies are necessary to provide more information about the mechanisms underlying the hypoglycemic, hypolipidemic and anti-inflammatory effects of beta-pinene in diabetic rats.

## Figures and Tables

**Table 1:** Evaluation of biochemical parameters in rat sera after oral (v.o.) treatment with *β*-pinene for 7 days.

Parameters	Vehicle —	Glib (5 mg/kg, v.o.)	Beta-pinene (mg/kg, v.o.)		
			25	50	100
Glucose (mg/dL)	334.1 ± 27.23 —	77.57 ± 21 ^*∗∗∗∗*^ (76.7%)	393.9 ± 12.21 —	126.3 ± 18.62 ^*∗∗∗∗*^ (62.2%)	140.4 ± 27.25 ^*∗∗∗∗*^ (57.9%)
CT (mg/dL)	88.07 ± 8.791 —	65.88 ± 5.721 ^*∗∗∗*^	78.82 ± 6.616 —	86.57 ± 5.694 —	82.17 ± 11.2 —
HDL (mg/dL)	182.6 ± 8.049 —	26.79 ± 6.284 ^*∗∗∗∗*^ (85.3%)	178.5 ± 18.68 —	64.57 ± 3.867 ^*∗∗∗∗*^ (64.6%)	46 ± 7.326 ^*∗∗∗∗*^ (74.8%)
LDL (mg/dL)	64.57 ± 3.867 —	12.93 ± 1.902 ^*∗∗∗∗*^ (79.9%)	58 ± 7.849 —	15.69 ± 2.948 ^*∗∗∗∗*^ (75.7%)	14.9 ± 2.241 ^*∗∗∗∗*^ (76.9%)
VLDL (mg/dL)	112.5 ± 17.31 —	14.72 ± 1.224 ^*∗∗∗∗*^ (86.9%)	85.82 ± 7.535 ^*∗∗∗*^ (23.7%)	15.92 ± 2.54 ^*∗∗∗∗*^ (85.8%)	17.08 ± 0.8871 ^*∗∗∗∗*^ (84.8%)
TG (mg/dL)	354 ± 22.88 —	65.88 ± 5.721 ^*∗∗∗∗*^ (81.3%)	422 ± 33.73 —	168.4 ± 26.02 ^*∗∗∗∗*^ (52.7%)	145.6 ± 23.96 ^*∗∗∗∗*^ (58.8%)
AST (UI/L)	88.68 ± 2.88 —	52.4 ± 6.54 ^*∗*^	67.08 ± 3.9 —	61.48 ± 4.39 —	61.6 ± 5.68 —
ALT (UI/L)	70.8 ± 5.01 —	38.6 ± 3.911 ^*∗*^	63.86 ± 4.17 —	52.7 ± 10.42 —	63.98 ± 11.18 —

Values represent mean ± standard error of the mean (S.E.P.M), (*n* = 10). The values were considered significant at ^*∗∗*^*p* < 0.01; ^*∗∗∗*^, *p* < 0.001; ^*∗∗∗∗*^, *p* < 0.0001 vs. vehicle. Analyzed through two-way ANOVA and followed by Bonferroni (*n* = 10 mice per group).

## Data Availability

The data used to support the findings of this study are available on request.
